# *Akkermansia muciniphila* Ameliorates Midlife Obesity-Induced Cognitive Impairment via the Microbiota-Metabolism-Neuroinflammation Axis

**DOI:** 10.3390/ijms27198771

**Published:** 2026-09-30

**Authors:** Xiaomei Bian, Jiaqi Cao, Anrui He, Weiran Jian, Wei Wang, Xu Zha

**Affiliations:** 1 Department of Physiology and Pathophysiology, School of Basic Medical Sciences, Laboratory for Clinical Medicine, Laboratory of Organ Synergy and Intelligent Regenerative Manufacturing, Capital Medical University, Beijing 100069, China; 1715023@ccmu.edu.cn (X.B.); cjq@mail.ccmu.edu.cn (J.C.); 2 School of Basic Medical Sciences, Capital Medical University, Beijing 100069, China; heanrui@mail.ccmu.edu.cn (A.H.); jianweiran@mail.ccmu.edu.cn (W.J.)

**Keywords:** *Akkermansia muciniphila*, midlife obesity, age-related cognitive decline, gut–brain axis, neuroinflammation, synaptic integrity

## Abstract

Midlife obesity is an established independent risk factor for age-related cognitive decline, yet direct causal experimental evidence and underlying gut microbial mechanisms remain poorly defined. Using a mouse model of high-fat diet (HFD)-induced obesity restricted to the midlife period (6–12 months of age) in male wild-type C57BL/6J mice, we characterized the temporal and causal relationship between midlife weight gain and cognitive deterioration, and investigated the mediating function of *Akkermansia muciniphila* (Akk). Longitudinal body weight tracking across the lifespan revealed a stage-specific dynamic pattern, with sustained adipose tissue accumulation occurring during the midlife period (6–12 months of age). Midlife HFD exposure triggered multi-dimensional cognitive deficits accompanied by significant depletion of intestinal Akk, while oral supplementation of Akk effectively attenuated HFD-induced weight gain and ameliorated spatial, recognition and contextual memory impairments. Mechanistically, Akk suppressed hippocampal glial hyperactivation and proinflammatory factor upregulation, attenuated neuronal loss, and preserved hippocampal synaptic integrity. Serum metabolomics further identified midlife-specific metabolic remodeling characterized by dysregulated lipid metabolism and depleted microbiota-derived indole metabolites. Our findings establish a causal link between midlife obesity and accelerated cognitive aging, and identify Akk as a promising early intervention target for age-related cognitive decline.

## 1. Introduction

Age-related cognitive decline constitutes a growing public health challenge amid global population aging. Progressive impairment of learning, memory, and executive function not only compromises quality of life in older adults but also serves as a robust prodromal predictor of neurodegenerative diseases, including Alzheimer’s disease [1]. Converging lines of epidemiological and preclinical evidence have consistently identified midlife obesity as an independent risk factor for age-related cognitive decline, with midlife adiposity shown to exacerbate cognitive impairment in later life [2,3]. Longitudinal population studies have demonstrated that elevated body mass index in midlife is associated with reduced hippocampal volume, impaired episodic memory, and worse executive performance in advanced age, and these associations remain significant after adjustment for late-life weight status [4]. Midlife represents a critical transitional window for brain aging: metabolic disturbances emerging during this stage can trigger sustained central neuroinflammation, synaptic dysfunction, and neuronal damage, accelerating the trajectory of cognitive decline, and such pathological impairments are largely irreversible once progressing to advanced age [5,6,7,8]. However, human observational studies are inherently limited by residual confounding and reverse causality, and direct longitudinal experimental evidence delineating the temporal relationship between midlife weight gain and the subsequent onset of cognitive decline remains insufficient.

The gut microbiome is widely acknowledged as a critical mediator of the gut–brain axis, influencing both metabolic homeostasis and central nervous system activity through diverse mechanisms [9,10,11,12]. Gut microbial dysbiosis contributes to the pathogenesis of obesity and modulates neuroinflammation and synaptic plasticity via circulating metabolites, immune signaling pathways, and neuroendocrine circuits, thereby influencing cognitive trajectories [13]. The commensal bacterium *Akkermansia muciniphila* (Akk) degrades mucin and resides within the intestinal mucus layer; its beneficial impact on metabolic homeostasis has been well-documented [14,15,16]. Accumulating studies have documented a consistent reduction in intestinal Akk abundance in both obesity and aging [17,18,19,20]. Supplementation with Akk ameliorates metabolic dysfunction and systemic low-grade inflammation in obese hosts, and emerging preclinical data indicate its neuroprotective potential across multiple neurological disease models [21]. Nevertheless, it remains unclear whether diminished intestinal Akk mediates the adverse impact of midlife obesity on late-life cognitive function, and the central and peripheral mechanisms underlying this putative association have not been systematically investigated.

To address these critical knowledge gaps, the present study was designed to characterize the temporal association between midlife weight gain and cognitive decline, and to investigate the mediating role of intestinal Akk and its underlying mechanisms. First, we characterized age-related dynamic changes in body weight and body composition via longitudinal lifespan monitoring, and established a midlife high-fat diet (HFD)-induced obesity mouse model (6–12 months of age) to examine the effects of midlife obesity on cognitive function and its correlation with intestinal Akk depletion. Second, we evaluated the protective effect of Akk supplementation against HFD-induced cognitive deficits. Finally, we explored the underlying mechanisms with respect to hippocampal neuroinflammation, neuronal and synaptic integrity, and serum metabolic profiles. This study provides preclinical experimental evidence for the gut–brain axis mechanism linking midlife obesity to accelerated cognitive aging, and identifies Akk as a promising microbial target for the early prevention of age-related cognitive decline.

## 2. Results

### 2.1. Midlife High-Fat Diet-Induced Obesity Accelerates Multi-Dimensional Cognitive Decline Alongside Age-Associated Body Composition Remodeling in Mice

1. Midlife high-fat diet-induced obesity accelerates multi-dimensional cognitive decline alongside age-associated body composition remodeling in mice.

To characterize the physiological trajectory of aging and establish a reliable midlife intervention paradigm, we first longitudinally monitored body weight in mice across the lifespan. Body weight exhibited a stage-specific dynamic pattern: rapid somatic growth from 2 to 5 months of age, a short growth plateau at 5–6 months, sustained slow elevation during the 6–12 months midlife window, and gradual age-related weight loss after 12 months (Figure 1A). Body-composition analysis revealed that 12-month-old mice had an 81.2% higher body-fat percentage than 3-month-old mice (3-month group: 14.89 ± 0.61%; 12-month group: 26.98 ± 0.80%; *p* < 0.0001). Conversely, the proportion of lean body mass (LBM) was 18.4% lower in 12-month-old mice than in 3-month-old mice (3-month group: 48.24 ± 0.36%; 12-month group: 39.36 ± 0.94%; *p* < 0.0001; Figure 1B). Thus, the increase in body weight during midlife was accompanied by increased adiposity and a corresponding reduction in the proportional contribution of lean mass.

Accumulating epidemiological evidence has confirmed midlife obesity as an independent risk factor for late-life cognitive decline, yet direct experimental evidence for their causal association and underlying pathological mechanisms remains limited. To address this scientific question, we established a midlife obesity mouse model via high-fat diet (HFD) feeding from 6 to 12 months of age (Figure 1C), and further investigated the impact of midlife obesity on cognitive function.

Body weight tracking demonstrated that 6 months of high-fat diet intervention initiated at 6 months of age significantly accelerated weight gain (*p* < 0.0001; Figure 1D), confirming the successful establishment of diet-induced obesity during midlife. We next systematically evaluated cognitive function across groups. In the acquisition phase of the Morris water maze (MWM) test, the learning curves of the 3-month-old and 12-month-old normal chow groups were comparable (*p* = 0.9014), whereas 12-month-old HFD-fed mice exhibited significantly longer escape latencies than age-matched controls (*p* < 0.0001; Figure 1E). In the spatial probe trial, the percentage of time spent in the target quadrant was comparable between the two control groups (3-month group: 32.11 ± 1.56%; 12-month group: 34.23 ± 2.43%; *p* = 0.7307). Compared to age-matched controls, HFD-fed mice spent 29.7% less time in the target quadrant (12-month group: 34.23 ± 2.43%; 12-month-old HFD group: 24.06 ± 1.82%; *p* = 0.0050; Figure 1F). Similarly, platform-site crossings were comparable between the two control groups (3-month group: 3.29 ± 0.42 crossings; 12-month group: 2.86 ± 0.59 crossings; *p* = 0.7788). Compared to age-matched controls, HFD-fed mice made 60.1% fewer crossings (12-month group: 2.86 ± 0.59 crossings; 12-month-old HFD group: 1.14 ± 0.26 crossings; *p* = 0.0362; Figure 1G). Representative swimming trajectories further corroborated these findings: 3 m and 12 m control mice adopted a clear goal-directed search strategy, whereas 12 m + HFD mice exhibited non-targeted roaming behavior (Figure 1H). Novel-object exploration was comparable between the two control groups (3-month group: 63.90 ± 3.39%; 12-month-old HFD group: 63.41 ± 3.08%; *p* = 0.9936). Compared to age-matched controls, HFD-fed mice exhibited a 21.9% reduction in novel-object exploration (12-month group: 63.41 ± 3.08%; 12-month-old HFD group: 49.52 ± 2.96%; *p* = 0.0156; Figure 1I), indicating impaired recognition memory. Contextual fear conditioning produced a similar pattern. Freezing time was comparable between the two control groups (3-month group: 68.55 ± 3.76%; 12-month group: 65.66 ± 7.96%; *p* = 0.9424). Compared to age-matched controls, HFD-fed mice exhibited 37.3% less freezing (12-month group: 65.66 ± 7.96%; 12-month-old HFD group: 41.13 ± 6.17%; *p* = 0.0306; Figure 1J). These findings indicate that midlife HFD exposure also impaired hippocampus-dependent contextual memory.

Collectively, these results demonstrate that under normal dietary conditions, core cognitive functions including spatial memory, recognition memory, and contextual memory remain largely preserved in mice at the midlife stage, which is consistent with previous findings [22]. Obesity induced by high-fat diet during midlife is sufficient to impair multi-dimensional cognitive performance, providing direct causal evidence that midlife obesity accelerates the progression of cognitive aging.

### 2.2. Akk Supplementation Attenuates Hfd-Induced Obesity and Multi-Dimensional Cognitive Decline in Middle-Aged Mice

Our findings suggest that midlife high-fat diet-induced obesity drives multi-dimensional cognitive decline. Accumulating studies have demonstrated that the abundance of Akk consistently decreases during both physiological aging and pathological obesity, suggesting its potential mediating role in the association between obesity and cognitive impairment.

To determine the effect of midlife HFD feeding on intestinal Akk abundance, we quantified fecal Akk using real-time PCR (qPCR). After normalization to the CD group, the relative abundance of Akk was markedly reduced from 1.000 ± 0.1640 in the CD group to 0.01679 ± 0.003014 in the HFD group, corresponding to an approximately 59.6-fold lower (*p* = 0.0001; Figure 2A), confirming HFD-associated Akk depletion. To investigate the regulatory effect of Akk on midlife-obesity-related cognitive decline, we administered live Akk via oral gavage to mice alongside high-fat diet feeding. Body weight monitoring further showed that Akk attenuated HFD-induced weight gain (*p* < 0.0001; Figure 2B). Akk abundance was approximately 68.4-fold higher in the HFD + Akk group than in the HFD group (0.78 ± 0.07 vs. 0.01 ± 0.01, *p* < 0.0001) and was comparable to that in the CD group (1.00 ± 0.13, *p* = 0.1908; Figure 2C), confirming successful Akk supplementation. Behavioral assessments demonstrated that Akk supplementation attenuated HFD-associated cognitive impairment. In the MWM, mice in the HFD group exhibited significantly longer escape latency than the CD group (*p* = 0.0164), whereas HFD + Akk mice showed markedly shorter escape latency than the HFD group (*p* = 0.0037; Figure 2D). In the probe trial, HFD feeding reduced the time spent in the target quadrant by 24.1% (CD group: 29.92 ± 2.18%; HFD group: 22.70 ± 1.80%; *p* = 0.0331) compared to the CD group. Compared with the HFD group, Akk supplementation increased the time spent in the target quadrant by 39.7% (HFD group: 22.70 ± 1.80%; HFD + Akk group: 31.72 ± 1.56%; adjusted *p* = 0.0076) and increased the median number of platform-site crossings from 1 to 3 [HFD group, 1 (1–1.75); HFD + Akk group, 3 (2–3); adjusted *p* = 0.0226; Figure 2E,F]. Similarly, Akk supplementation increased novel-object exploration by 50.3% (HFD group: 46.82 ± 3.83%; HFD + Akk group: 70.38 ± 5.51%; *p* = 0.0054; Figure 2G) and contextual freezing by 67.5% (HFD group: 49.79 ± 6.25%; HFD + Akk group: 83.41 ± 2.58%; *p* = 0.0003; Figure 2H), indicating protection against HFD-associated recognition and contextual memory impairment.

Collectively, these results demonstrate that Akk supplementation alleviates high-fat diet-induced obesity and reverses multi-dimensional cognitive impairment in middle-aged mice, directly identifying Akk as a key gut microbial mediator of obesity-related cognitive decline during midlife.

### 2.3. Neuroinflammation Is a Key Pathway Underlying Akk-Mediated Cognitive Improvement in Obesity

Since our results suggest that reduced intestinal Akk abundance mediates the exacerbation of cognitive decline by midlife obesity, we further investigated the underlying mechanisms focusing on neuroinflammatory pathways. As the core brain region governing spatial learning and memory, the hippocampus undergoes robust glial activation and proinflammatory factor accumulation in the context of obesity-associated cognitive impairment. We therefore systematically evaluated the regulatory effects of Akk on hippocampal neuroinflammation in HFD-fed middle-aged mice at three levels: glial cell activation status, inflammatory cytokine transcription, and protein expression.

Immunofluorescence staining was performed to label ionized calcium-binding adapter molecule 1 (Iba1, a specific microglial marker) and glial fibrillary acidic protein (GFAP, a specific astrocyte marker), respectively, to assess glial activation in the CA1 region of the hippocampus. All quantified values were normalized to the CD group. Compared with controls, HFD increased the normalized Iba1-positive area, a marker of microglial activation, to 1.87 ± 0.14-fold of the CD level (*p* = 0.0002), whereas Akk supplementation reduced it to 1.17 ± 0.09-fold (*p* = 0.0013 vs. HFD; Figure 3A,B). Similarly, the normalized GFAP-positive area increased to 1.59 ± 0.05-fold following HFD feeding (*p* = 0.0015) and decreased to 1.03 ± 0.12-fold following Akk supplementation (*p* = 0.0022 vs. HFD; Figure 3C,D), indicating attenuated astrocyte activation.

All mRNA expression levels were normalized to those in the CD group. Compared with the CD group, HFD feeding increased the hippocampal mRNA expression levels of IL-6 (4.99 ± 0.96-fold, *p* = 0.0010), IL-1β (3.39 ± 0.48-fold, *p* = 0.0003), TNF-α (3.99 ± 0.55-fold, *p* = 0.0001), CCL2 (1.71 ± 0.20-fold, *p* = 0.0211), and CCL5 (1.68 ± 0.07-fold, *p* = 0.0036). Compared with the HFD group, Akk supplementation reduced the corresponding expression levels to 0.94 ± 0.15-fold (*p* = 0.0009), 1.38 ± 0.17-fold (*p* = 0.0014), 0.98 ± 0.11-fold (*p* < 0.0001), 0.94 ± 0.04-fold (*p* = 0.0132), and 1.00 ± 0.11-fold (*p* = 0.0007), respectively (Figure 3E–I).

All protein expression levels were normalized to the corresponding loading controls and subsequently expressed relative to those in the CD group. Compared to the CD group, HFD feeding increased the hippocampal protein expression levels of IL-1β (2.13 ± 0.33-fold, *p* = 0.0148), IL-6 (1.85 ± 0.10-fold, *p* = 0.0029), TNF-α (3.50 ± 0.50-fold, *p* = 0.0040), and iNOS (2.35 ± 0.45-fold, *p* = 0.0297). Compared to the HFD group, Akk supplementation reduced the corresponding protein levels to 0.80 ± 0.22-fold (*p* = 0.0071), 0.83 ± 0.15-fold (*p* = 0.0011), 1.66 ± 0.14-fold (*p* = 0.0175), and 0.88 ± 0.13-fold (*p* = 0.0206), respectively Figure 3J,K).

Collectively, these data indicate that Akk alleviates HFD-induced hippocampal neuroinflammation by suppressing excessive glial activation and downregulating the transcription and protein expression of proinflammatory factors. Our findings suggest that inhibition of neuroinflammation represents an important mechanism by which Akk improves cognitive function in HFD-fed middle-aged mice.

### 2.4. Akk Supplementation Attenuates HFD-Induced Neuronal Loss and Restores Synaptic Integrity in Middle-Aged Mice

We further investigated the underlying structural mechanisms, given that neuronal loss and synaptic damage are well-established pathological hallmarks of inflammation-driven cognitive decline. Mature neurons were labeled with neuronal nuclei (NeuN) immunofluorescence staining to quantify neuronal density in the cerebral cortex and hippocampal subregions (CA1, CA3, and dentate gyrus, DG). Synaptic integrity was assessed by measuring the expression of synapse-associated proteins in the hippocampus via Western blot.

NeuN immunostaining showed that HFD reduced the normalized densities of NeuN-positive neurons in the cerebral cortex (0.49 ± 0.04-fold, *p* < 0.0001), CA1 (0.63 ± 0.03-fold, *p* = 0.002), CA3 (0.46 ± 0.03-fold, *p* < 0.0001), and DG (0.78 ± 0.04-fold, *p* = 0.0233) relative to the CD group. Akk supplementation attenuated these reductions in the cortex (0.84 ± 0.04-fold, *p* = 0.0025), CA1 (1.08 ± 0.09-fold, *p* = 0.0004), CA3 (0.99 ± 0.04-fold, *p* < 0.0001), and DG (1.00 ± 0.03-fold, *p* = 0.0213) compared with the HFD group (Figure 4A,B). Consistently, HFD significantly reduced hippocampal expression of SYN1 (0.30 ± 0.02-fold, *p* = 0.0058), CaMKIIα (0.52 ± 0.07-fold, *p* = 0.0017), and SYP (0.41 ± 0.05-fold, *p* = 0.0006) relative to the CD group. Akk supplementation attenuated the HFD-induced reductions in SYN1, CaMKIIα, and SYP, with expression levels of 0.96 ± 0.10-fold (*p* = 0.0080), 0.85 ± 0.04-fold (*p* = 0.0107), and 0.93 ± 0.06-fold (*p* = 0.0013), respectively, compared with the HFD group. PSD95 expression did not differ significantly between the CD and HFD groups (1.00 ± 0.08-fold vs. 0.73 ± 0.05-fold; *p* = 0.1106) and showed a nonsignificant increasing trend following Akk supplementation (1.17 ± 0.30-fold vs. HFD, *p* = 0.0560; Figure 4C,D).

Collectively, these data demonstrate that midlife HFD exposure induces neuronal loss and disrupts synaptic integrity across multiple brain regions, whereas Akk supplementation effectively attenuates this neuronal damage. This neuroprotective effect likely underpins the cognitive improvements observed in Akk-treated obese mice.

### 2.5. Serum Metabolic Profiles Differ Markedly Between Young and Middle-Aged Mice

We have defined the central mechanisms by which Akk mediates midlife obesity-induced cognitive decline. As an intestinal symbiont, Akk cannot directly act on the central nervous system, with circulating metabolites serving as critical gut–brain axis mediators. To explore the peripheral metabolic basis, we performed untargeted serum metabolomics across young and middle-aged mice.

Unsupervised principal component analysis (PCA) revealed distinct serum metabolic profiles between young and middle-aged mice. The first principal component (PC1) and second principal component (PC2), which represent orthogonal linear combinations of the measured metabolic features rather than predefined biological factors, explained 37.25% and 19.48% of the total variance, respectively, accounting for a cumulative variance of 56.73% (Figure 5A). Distinct intergroup separation with tight intragroup clustering was observed between young and middle-aged mice, indicating age as a core driver of serum metabolic characteristics (Figure 5A). Supervised partial least squares-discriminant analysis (PLS-DA) with model parameters R^2^Y = 0.98 and Q^2^Y = 0.43 (no severe overfitting) further confirmed non-overlapping 95% confidence ellipses between the two groups (Figure 5B). Using VIP > 1 and *p* < 0.05 as screening thresholds, we identified differentially abundant metabolites that formed two distinct expression clusters. The cluster upregulated in middle-aged mice was dominated by free fatty acids (linoleic acid, palmitoleic acid, oleic acid) and glycerophospholipids (PE, PG), while the downregulated cluster included gut microbiota-derived 3-(2-hydroxyethyl) indole, azelaic acid, and neurosteroid pregnenolone. Sample-level clustering showed no intergroup overlap, consistent with multivariate statistical results and supporting midlife-specific serum metabolic remodeling (Figure 5C).

Integration of these metabolomic findings with the established functions of Akk reveals that midlife metabolic remodeling aligns closely with the bacterium’s regulatory profile. In terms of lipid metabolism, elevation in circulating free fatty acids and glycerophospholipids reflects a lipid dysregulation phenotype that Akk is documented to reverse by modulating intestinal lipid absorption and hepatic metabolism [23]. Excess free fatty acids can cross the blood–brain barrier to trigger hippocampal neuroinflammation, which aligns with our observation that Akk supplementation suppressed glial hyperactivation and proinflammatory factor upregulation, suggesting improved circulating lipid profiles as a key peripheral mechanism underlying Akk’s anti-neuroinflammatory effect [24]. For microbiota-derived metabolites, reduced indole derivatives in middle-aged mice indicate impaired microbial tryptophan metabolism. As canonical aryl hydrocarbon receptor (AhR) agonists that maintain intestinal barrier integrity and suppress systemic inflammation [25,26], indole metabolites can be promoted by Akk via regulating intestinal tryptophan catabolism [27]. Combined with our finding that Akk ameliorated cognitive impairment and preserved neuronal and synaptic integrity, these data suggest that Akk exerts neuroprotective effects by boosting indole metabolites to attenuate peripheral inflammation and neurotoxic insults, preliminarily supporting the mediating role of the microbiota–metabolism–neuroinflammation axis.

## 3. Discussion

In the present study, we systematically characterized the longitudinal trajectory of body weight across the mouse lifespan. Using a midlife high-fat diet-induced obesity model, we further confirmed that midlife obesity accelerates cognitive aging, which is accompanied by a significant reduction in intestinal Akk abundance. Supplementation with Akk effectively ameliorated multi-dimensional cognitive deficits in obese middle-aged mice, and the underlying mechanisms involved the suppression of hippocampal glial activation and neuroinflammation, as well as the preservation of neuronal survival and synaptic integrity. Furthermore, serum metabolomics analysis revealed age-associated metabolic remodeling characterized by dysregulated lipid metabolism and depleted microbiota-derived indole metabolites at midlife, which may serve as critical peripheral mediators linking intestinal Akk to central cognitive function. Collectively, our findings provide experimental evidence supporting a causal effect of midlife obesity on accelerated cognitive decline and identify Akk as a promising target for early microbiome-targeted intervention. The potential involvement of a microbiota–metabolism–neuroinflammation axis requires further investigation.

Accumulating epidemiological evidence has consistently linked midlife obesity to an increased risk of late-life dementia and cognitive impairment [6,28]. However, most human studies are limited to cross-sectional or retrospective observational designs, which can only establish correlation rather than causation, and are confounded by heterogeneous genetic, lifestyle, and comorbidity factors. In this study, we employed a longitudinal mouse model with strictly controlled genetic background and environmental conditions, and revealed a clear temporal sequence: midlife (6–12 months) is characterized by progressive fat accumulation and preserved cognitive function. More importantly, high-fat diet intervention specifically during the midlife period was sufficient to induce overt cognitive deficits as early as 12 months of age, directly demonstrating that midlife obesity causally accelerates the progression of cognitive aging. These findings provide preclinical causal evidence supporting the epidemiological association, and reinforce the notion that midlife represents a critical window for intervention to delay cognitive decline.

Our study further identified Akk as a key microbial mediator linking midlife obesity to cognitive deterioration. Akk is a well-recognized beneficial intestinal symbiont whose abundance declines with both obesity and aging [18,23,29,30,31]. Previous preclinical studies have reported the neuroprotective effects of Akk in aged mice and Alzheimer’s disease models [32,33], but its role in midlife obesity-associated cognitive impairment remained largely unexplored. Here we showed that high-fat diet-induced obesity in midlife caused a marked reduction in intestinal Akk, and that supplementation with Akk via gavage not only attenuated weight gain but also significantly improved spatial memory, recognition memory, and contextual memory in obese middle-aged mice. These findings extend the current understanding of Akk’s beneficial effects from metabolic disorders to cognitive function, and highlight its translational potential as an early microbiome-targeted intervention for midlife obesity-related cognitive aging. Notably, our study focused on intervention during the midlife stage, which is more clinically relevant for primary prevention of dementia compared with interventions initiated at advanced ages when neuronal damage is largely irreversible.

At the central level, our findings revealed that suppression of hippocampal neuroinflammation and preservation of neuronal and synaptic integrity represent core mechanisms underlying the cognitive benefits of Akk. Chronic neuroinflammation, driven by excessive activation of microglia and astrocytes and sustained release of proinflammatory cytokines, is a well-documented pathological hallmark of obesity-associated cognitive decline [34,35,36]. In line with previous reports, we observed that midlife high-fat diet induced robust activation of Iba1-positive microglia and GFAP-positive astrocytes in the hippocampus, accompanied by elevated transcription and protein expression of multiple proinflammatory factors including IL-1β, IL-6, TNF-α, and iNOS. Excessive proinflammatory milieu in the hippocampus is known to impair synaptic plasticity, induce neuronal apoptosis, and ultimately lead to cognitive dysfunction. Importantly, Akk supplementation effectively attenuated glial overactivation and downregulated the inflammatory cascade, which in turn mitigated neuronal loss and attenuated the HFD-induced reduction in the expression of synaptic proteins such as SYN1, CaMKIIα, and SYP. These results are consistent with the established anti-inflammatory properties of Akk, and provide a structural basis for its cognitive protective effects at the hippocampal level.

At the peripheral level, our metabolomics data provide mechanistic insights into how an intestinal bacterium modulates brain function via circulating metabolic mediators. First, we found that middle-aged mice already exhibited systemic lipid metabolic dysregulation, with elevated levels of free fatty acids and glycerophospholipids. It is well established that Akk regulates intestinal lipid absorption and hepatic lipid metabolism and may thereby influence circulating lipid levels, while excess circulating free fatty acids can cross the blood–brain barrier and promote microglial activation and neuroinflammation [23,37,38]. Based on these previous findings, we hypothesize that modulation of circulating lipid profiles may represent one potential pathway through which Akk attenuates central inflammation and cognitive impairment [39,40]. However, because serum metabolomics was not performed in the HFD + Akk group in the present study, this possibility remains speculative and requires direct experimental validation. Second, we identified the gut microbiota-derived indole metabolite 3-(2-hydroxyethyl) indole as a key molecule downregulated in middle-aged mice. Tryptophan-derived indole metabolites are classic AhR agonists that maintain intestinal barrier integrity and suppress systemic inflammation, and their depletion has been closely associated with neuroinflammation and cognitive dysfunction [41,42,43]. Emerging evidence indicates that Akk can modulate intestinal tryptophan catabolism to promote the production of indole derivatives [27]. Taken together, these observations raise the possibility that Akk may support intestinal barrier function and reduce peripheral inflammation through the modulation of microbiota-derived indole metabolites. However, because metabolomic profiling was not performed in the HFD and HFD + Akk groups, whether these metabolites are directly modulated by Akk and mediate its cognitive benefits remains to be determined. Further targeted metabolomic analyses and mechanistic intervention studies are required to evaluate this proposed pathway.

From a translational perspective, several strategies may potentially support the maintenance or enrichment of intestinal Akk in humans. Dietary approaches that increase the availability of microbiota-accessible substrates, including fermentable dietary fiber, prebiotic components, and polyphenol-rich foods, have been suggested to favor a gut microbial environment compatible with Akk persistence or expansion [44,45,46]. In addition, direct supplementation with live or pasteurized Akk has emerged as a potential microbiome-targeted intervention, with recent clinical studies supporting its feasibility and metabolic benefits in individuals with overweight or obesity [47]. Nevertheless, whether dietary modulation or direct Akk supplementation can sustainably maintain intestinal Akk abundance and, more importantly, translate into long-term protection against cognitive decline in humans remains unknown. Prospective longitudinal studies and randomized controlled trials incorporating both microbiome and cognitive outcomes will therefore be required before Akk-targeted strategies can be recommended for the prevention of age-related cognitive impairment.

Several limitations of this study should be acknowledged. First, our serum metabolomics analysis was only performed in young and middle-aged mice on a normal chow diet, without including the HFD and Akk supplementation groups. Thus, we cannot directly demonstrate that Akk modulates HFD-induced metabolic disorders via the identified lipid and indole pathways; future metabolomic analysis of all intervention groups is required to validate the causal role of these metabolic mediators. Second, the mechanistic conclusions are largely based on correlational evidence. Further studies using germ-free mice, AhR inhibitors, or specific pathway knockout models are needed to establish a definitive causal link between Akk, indole metabolites, and cognitive improvement. Third, this study was conducted exclusively in male mice; whether the findings can be generalized to female mice requires further investigation given the known sex differences in metabolic and cognitive aging trajectories.

In conclusion, our study demonstrates that midlife obesity causally accelerates cognitive aging, and that Akk exerts protective effects against obesity-related cognitive decline by inhibiting hippocampal neuroinflammation and preserving neuronal and synaptic integrity. Age-associated dysregulation of lipid metabolism and depletion of microbiota-derived indole metabolites may constitute the peripheral metabolic basis of this process. These findings advance our understanding of the gut–brain axis mechanisms underlying cognitive aging, and support Akk as a promising early microbiome-targeted intervention strategy for delaying the onset of late-life cognitive decline.

## 4. Materials and Methods

### 4.1. Animal Studies

Male wild-type C57BL/6J mice aged 3, 6, and 12 months were purchased from Vital River Laboratory. All animals were specific pathogen-free (SPF) and had not undergone any experimental procedures before enrollment. Mice were housed in a well-ventilated room maintained at 22 ± 3 °C and 40–60% relative humidity under a 12 h light/dark cycle (lights on from 07:00 to 19:00), with nesting material provided for environmental enrichment. Animals had ad libitum access to their assigned diets and drinking water, except that food was withheld during the fasting period specified for body composition assessment. Following arrival, mice were allowed to acclimatize to the animal facility for one week before experimental procedures. On each behavioral testing day, animals were transferred to the testing room in their home cages and allowed to acclimatize for 1 h before testing. All procedures were performed by trained personnel, with gentle handling and minimal unnecessary disturbance to reduce distress and stress. General health and welfare were assessed daily by monitoring activity, posture, grooming, and the ability to access food and water. Additional observations were conducted following oral gavage and behavioral testing. Body weight was monitored regularly, and a 10% reduction in body weight within one week was predefined as a humane endpoint requiring euthanasia. No adverse events were observed during the study.

Age-related differences in body composition and serum metabolomic profiles were assessed by comparing 3- and 12-month-old mice fed a chow diet (CD). To evaluate the effects of midlife high-fat diet (HFD) exposure on cognition, comparisons were performed among 3-month-old CD-fed controls, 12-month-old CD-fed controls, and 12-month-old HFD-fed mice. The effects of Akk supplementation were examined in age-matched CD, HFD, and HFD + Akk groups. For the dietary-intervention experiments, mice of the same age were randomly allocated to the control and treatment groups using a random number table. For age-related comparisons, mice were grouped according to their chronological age, without random allocation to age groups. Sample sizes were determined with reference to published studies and previous experimental experience in our laboratory, consistent with the principles of replacement, reduction, and refinement (3Rs). To minimize potential confounding, housing conditions and handling procedures were standardized across groups. Treatments and measurements were conducted within consistent daily time windows, and the order in which animals underwent these procedures was randomized across groups. Cage positions within the animal facility were also randomly assigned. Investigators responsible for group allocation, dietary interventions, and gavage administration were aware of group assignments. Outcome assessment and statistical analysis were performed by investigators blinded to group allocation using coded animal and sample identifiers. Group identities were disclosed only after outcome assessment and statistical analysis had been completed.

A total of 99 mice were included in the study and were assigned to three experimental cohorts. The first cohort comprised 29 mice used for longitudinal monitoring of body weight across the lifespan. The second cohort comprised 16 mice used to compare body composition between 3 and 12 months of age. The third cohort comprised 54 mice used for the midlife dietary-intervention experiments. The baseline body weights of the animals in the two experimental cohorts were as follows. In the first experiment evaluating the effects of midlife HFD exposure, mice were assigned to the control diet (CD: 32.51 ± 1.21 g) or high-fat diet (HFD: 32.74 ± 1.90 g) group. In the second experiment evaluating the effects of Akk supplementation, mice were assigned to the CD group (33.17 ± 2.29 g), HFD group (34.20 ± 2.53 g), or HFD + Akk group (33.09 ± 1.60 g). No significant differences in baseline body weight were observed among the experimental groups within either experiment (*p* > 0.05). All animal experimental procedures were reviewed and approved by the Animal Ethics Committee of Capital Medical University (approval no. AEEI-2025-611; approval date: 15 June 2025) and were conducted in accordance with the Guide for the Care and Use of Laboratory Animals. The research questions, experimental design, and analysis plan were established before the study commenced. The study protocol was not prospectively registered. No animals, experimental units, or data points were excluded from the analyses.

Mice were allocated to receive either standard chow or a high-fat diet with 60% fat content for 6 consecutive months. The HFD (MP Biomedicals) provided 60% of total energy from fat, 20% from protein, and 20% from carbohydrates; lard and soybean oil were the principal fat sources. The detailed composition of each 10 kg of the diet is provided in Table 1. The 6-month exposure was used to model sustained HFD consumption across the murine midlife window and its cumulative neurobehavioral effects [48,49]. Body weight measurements were documented for all mice at monthly intervals.

#### 4.1.1. TD-NMR-Based Body Composition Analysis

Body composition of awake mice was determined non-invasively via a time-domain nuclear magnetic resonance (TD-NMR) instrument (EchoMRI-100H, Echo Medical Systems, Houston, TX, USA), which enables separate quantification of whole-body fat, lean tissue, total water, and free fluid based on distinct relaxation patterns of hydrogen protons across various tissues. Prior to scanning, animals underwent a 4–6 h fasting period with free access to water, and body weight was recorded to 0.01 g. Each mouse was gently positioned inside a cylindrical restraint tube to ensure complete placement within the detection coil, followed by a standard 90-s measurement protocol. Two consecutive scans were conducted per animal, and the averaged values were used for statistical analysis. Fat and lean percentages were derived by dividing the respective tissue mass by total body weight.

#### 4.1.2. Morris Water Maze Test

Before the start of the formal test, the mice were habituated to the testing room and apparatus. The experiment ran for six consecutive days, with the initial five days dedicated to acquisition training. In each training session, animals were released from four different start points, always facing the pool wall. The latency to find and mount the submerged platform was measured as the escape latency. Any mouse that did not locate the platform within the prescribed cutoff time was manually guided to it by the investigator. On the sixth day, a probe trial was carried out with the platform removed. Mice were again introduced into the water facing the wall, and two parameters were assessed: the proportion of total time spent in the target quadrant (the previous platform location) and the number of times the animal crossed the exact site where the platform had been positioned.

#### 4.1.3. Novel Object Recognition Test

The procedure was divided into two stages: a familiarization phase and a testing phase. In the familiarization stage, each animal was introduced into an open-field arena with two identical objects and given 10 min to explore freely. One hour following the familiarization session, one of the two familiar items was exchanged for a new, unfamiliar object. The mouse was then placed back into the arena for another free-exploration period, during which the time spent investigating the novel object and the familiar object was measured separately.

#### 4.1.4. Contextual Fear Conditioning Test

The experiment consisted of two phases: the habituation phase and the contextual test phase. On day 1 (habituation phase), mice were placed in a fear conditioning chamber and allowed to acclimate. On day 2, mice were reintroduced to the identical fear conditioning chamber, and the percentage of freezing time throughout the test session was quantified.

#### 4.1.5. Cultivation of Akk and Mouse Colonization with Akk

The strain *Akkermansia muciniphila* MucT (ATCC BAA835) was propagated in brain–heart infusion (BHI) medium under oxygen-free conditions at 37 °C, following the protocol described earlier [50]. Cells harvested during the logarithmic growth phase were rinsed with BHI, to obtain a concentration of 1.5 × 10^9^ cells/mL [51]. This bacterial preparation, suspended in BHI, was delivered to mice by oral gavage on three days each week. For the control cohort, animals received sterile BHI broth alone as the vehicle.

#### 4.1.6. Western Blotting

Mouse hippocampal tissues were homogenized in a protein extraction buffer. Total protein concentration was determined using a BCA kit (Thermo Fisher Scientific, Waltham, MA, USA). After quantification of protein content, the samples were combined with SDS-PAGE loading buffer and heat-denatured in a heating block. Aliquots containing 30 μg of total protein per lane were resolved by SDS-PAGE and then electroblotted onto PVDF membranes. The membranes were first blocked with 5% (*w*/*v*) non-fat dry milk, followed by incubation with primary antibodies overnight at 4 °C. The primary antibodies used were as follows: anti-TNFα (1:1000), anti-IL-1β (1:1000), anti-iNOS (1:1000), anti-PSD95 (1:1000), anti-SYN1 (1:1000), anti-SYP (1:1000), anti-SNAP-25(1:1000), anti-β-tubulin (1:5000) and anti-GAPDH (1:5000). After the overnight primary antibody incubation, the PVDF membranes were incubated with the appropriate horseradish peroxidase (HRP)-conjugated secondary antibodies and subsequently visualized using a chemiluminescence imaging system (Tanon, Shanghai, China). Band intensities were quantified using ImageJ software (version 1.53q), and the expression level of each target protein was normalized to the corresponding β-tubulin/GAPDH band intensity from the same sample. The normalized values were subsequently expressed relative to those of the control group. Detailed information on all antibodies used in this study is provided in Table 2.

#### 4.1.7. Immunofluorescence Staining

Brain samples harvested from mice were subjected to fixation, dehydration, and embedding in OCT compound, and then cut into 40 μm frozen sections on a cryostat. These sections were rinsed three times with PBS, subsequently permeabilized by exposure to 0.3% Triton X-100 for 20 min, and then blocked using 2% BSA for 1 h. Following blocking, the sections were incubated overnight at 4 °C with primary antibodies directed against NeuN (1:1000), Iba1 (1:1000), and GFAP (1:1000). After three PBS washes, the samples were treated with an Alexa Fluor 488/594-conjugated secondary antibody (1:2000) for 1 h at room temperature. Following three further washes, nuclear counterstaining was performed with DAPI for 10 min. Finally, the sections were mounted on slides and examined under a fluorescence microscope. Detailed information on all antibodies used in this study is provided in Table 2.

#### 4.1.8. Quantitative PCR for Inflammatory Cytokines Analysis

Total RNA was extracted from mouse hippocampal tissue using an RNA isolation kit. RNA concentration and purity were evaluated using Nanodrop. A total of 1 μg RNA was reverse-transcribed using HiScript II One Step RT-PCR Kit. Quantitative PCR was performed using qPCR SYBR Green Master Mix on an ABI StepOne Plus instrument. The thermal cycling program consisted of an initial holding stage at 95 °C for 20 s, followed by 40 cycles of denaturation at 95 °C for 3 s and combined annealing/extension at 60 °C for 30 s. Melt-curve analysis was performed to confirm amplification specificity. Relative expression was calculated using the 2^−ΔΔCt^ method, with GAPDH as the reference gene and the CD group as the calibrator. The primer sequence is IL-1β-F: 5′-CTGCAGCTGGAGAGTGTGG-3′ and IL-1β-R: 5′-GGGGAACTCTGCAGACTCAA-3′, IL-6-F: 5′-AGTTGCCTTCTTGGGACTGA-3′ and IL-6-R: 5′-TCCACGATTTCCCAGAGAAC-3′, TNF-α-F: 5′-ACTGAACTTCGGGGTGATCG-3′ and TNF-α-R: 5′-GGTGGTTTGTGAGTGTGAGG-3′, CCL2-F: 5′-CCTGCTGCTACTCATTCACCA-3′ and CCL2-R: 5′-ATTCCTTCTTGGGGTCAGCA-3′, CCL5-F: 5′-GTGCCCACGTCAAGGAGTAT-3′ and CCL5-R: 5′-CTCTGGGTTGGCACACACTT-3′, Gapdh-F: 5′-AGGTCGGTGTGAACGGATTTG-3′; and Gapdh-R: 5′-TGTAGACCATGTAGTTGAGGTCA-3′.

#### 4.1.9. Quantitative PCR for Microbiota Analysis

Following microbiota colonization, fecal samples were harvested from mice, followed by genomic DNA (gDNA) extraction. The concentration of the extracted gDNA was subsequently normalized to 1 ng/mL. The abundance of target intestinal bacterial taxa was determined using PowerUp SYBR Green Master Mix in combination with group-specific primers targeting the 16S rRNA gene. Total Eubacteria 16S rDNA was served as an internal control. The primer sequence is Akk-F: 5′-CAGCACGTGAAGGTGGGGAC-3′ and Akk-R: 5′-CCTTGCGGTTGGCTTCAGCAG-3′, Eub-F: 5′-ACTCCTACGGGAGGCAGCAGT-3′ and Eub-R: 5′-ATTACCGCGGCTGCTGGC-3′

#### 4.1.10. Untargeted Metabolomics

All metabolite extraction and detection assays were performed by Novogene Co., Ltd. (Beijing, China). For sample pretreatment, pre-chilled 80% methanol was added to serum samples, followed by thorough vortex blending. The supernatant was harvested via centrifugation, then diluted with ultrapure water to adjust the methanol concentration to 53%. After a second round of centrifugation, the resulting supernatant was collected for subsequent UHPLC-MS/MS analysis. Chromatographic separation was conducted on a Hypesil Gold column at a constant flow rate of 0.2 mL/min. Mobile phase A consisted of 0.1% formic acid (FA) for positive ionization mode or 5 mM ammonium acetate for negative ionization mode, while mobile phase B was methanol. The gradient elution program was set as follows: 2% phase B was maintained for the initial 1.5 min; phase B was linearly elevated from 2% to 100% over 3 min; 100% B was held isocratically for 10 min; the proportion of B was then linearly reduced back to 2% within 10.1 min; finally, the column was re-equilibrated with 2% B for 12 min. For mass spectrometric detection, the spray voltage was set at 3.5 kV. The instrument parameters were configured as follows: sheath gas flow rate of 35 psi, capillary temperature of 320 °C, S-lens RF level of 60, auxiliary gas flow rate of 10 L/min, and auxiliary gas heater temperature of 350 °C. Metabolite features were annotated by matching the acquired mass spectrometric data against the metabolite databases used in the Novogene untargeted metabolomics workflow. Because database-based annotation in untargeted metabolomics is putative unless supported by sufficient structural evidence, automatically assigned compound names were not regarded as definitive metabolite identifications. Following re-evaluation of the annotation results, compounds with annotations suggestive of exogenous synthetic or medicinal-chemistry compounds and lacking biological plausibility under the present experimental conditions were excluded from the curated metabolite list prior to hierarchical clustering analysis.

Principal component analysis (PCA) was performed using metaX software (version 2.0.0) to visualize overall variation and clustering patterns among the serum metabolic profiles. Partial least squares-discriminant analysis (PLS-DA) was subsequently performed using metaX as a supervised approach to evaluate the separation between experimental groups. The goodness of fit and predictive performance of the PLS-DA model were assessed using the R^2^Y and Q^2^Y values, respectively. Variable importance in projection (VIP) scores were derived from the PLS-DA model. Metabolite features were considered differentially abundant when they met all of the following criteria: VIP > 1, *p* < 0.05, and fold change (FC) ≥ 2 or FC ≤ 0.5. Hierarchical clustering heatmaps were generated using the R package (version 4.6.1) “pheatmap” based on the curated metabolite dataset.

#### 4.1.11. Statistical Analysis

Normally distributed data are presented as the mean ± standard error of the mean (SEM), whereas non-normally distributed data are presented as the median with interquartile range (IQR). Individual biological observations are shown whenever applicable, with each data point representing one independent animal unless otherwise specified. Normality was assessed using the Shapiro–Wilk test together with visual inspection of Q–Q plots. A Shapiro–Wilk *p* value > 0.05, together with no substantial deviation from linearity in the Q–Q plot, was considered to indicate no significant departure from normality. Homogeneity of variance was evaluated using the Brown–Forsythe test.

Normally distributed data satisfying the assumption of equal variance were analyzed using an unpaired two-tailed Student’s t-test for two-group comparisons or one-way or two-way analysis of variance (ANOVA), as appropriate. When the assumption of equal variance was violated, Welch’s correction was applied. Non-normally distributed data were analyzed using the Mann–Whitney U test for two-group comparisons or the Kruskal–Wallis test followed by Dunn’s multiple-comparisons test for comparisons involving three or more groups. A significance level of *p* < 0.05 was adopted for all datasets. In the figures, significance is indicated as follows: ns, not significant; * *p* < 0.05; ** *p* < 0.01; *** *p* < 0.005; *** *p* < 0.001. Exact *p* values are reported in the main text whenever available. The statistical tests and biological sample sizes used for individual analyses are specified in the corresponding figure legends. Every experiment was performed independently at least three times. Statistical analyses of the data were performed using GraphPad Prism 8.0.

The sample size (n) represents the number of individual animals and therefore corresponds to biological replicates. Technical replicates, including repeated measurements of the same biological sample, were not treated as independent observations. When multiple technical measurements, tissue sections, or microscopic fields were obtained from the same animal, these values were averaged to generate a single value for that animal before statistical analysis. All statistical analyses were performed using biological replicates only. The exact number of animals included in each analysis is specified in the corresponding figure legend.

## Figures and Tables

**Figure 1:**
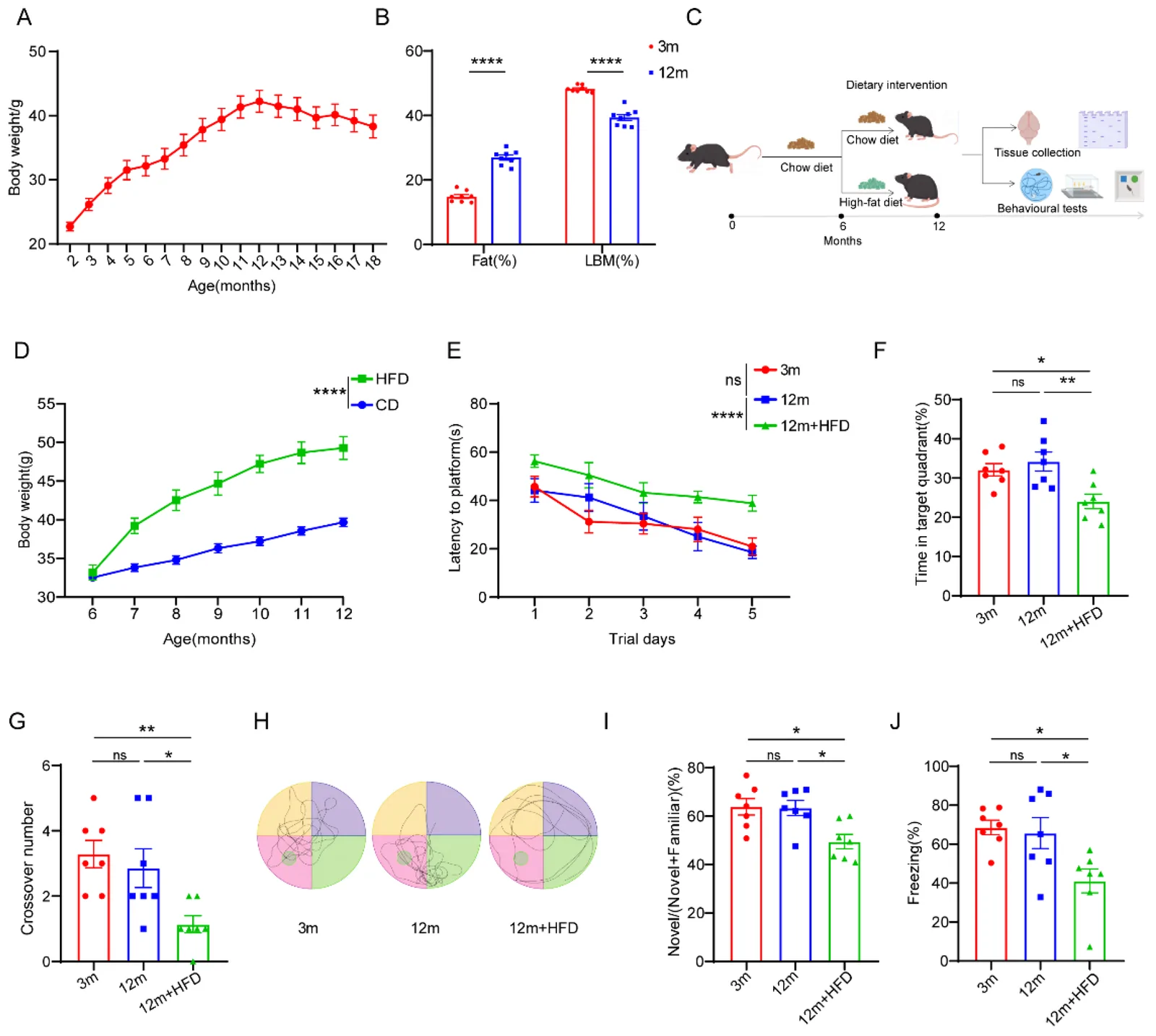
Longitudinal body composition dynamics and cognitive function in mice with midlife high-fat diet intervention. (**A**) Longitudinal changes in body weight of mice from 2 to 18 months of age. *n* = 29. (**B**) Quantification of body fat percentage (Fat%) and lean body mass percentage (LBM%) in 3-month-old (3 m) young adult mice and 12-month-old (12 m) middle-aged mice. *n* = 8 per group. (**C**) Schematic illustration of the experimental design. Mice were fed a normal chow diet (CD) or high-fat diet (HFD) during the midlife window (6 to 12 months of age), followed by behavioral tests and tissue collection. (**D**) Body weight curves of CD-fed and HFD-fed mice throughout the 6-month midlife intervention. *n* = 7 per group. (**E**) Escape latency across the 5-day acquisition phase of the MWM test in three groups. (**F**,**G**) Quantification of the percentage of time spent in the target quadrant (**F**) and the number of platform crossings (**G**) during the MWM spatial probe trial. *n* = 7 per group. (**H**) Representative swimming trajectories of mice from the three groups in the MWM probe trial. (**I**) Quantification of the novel object exploration time percentage in the NOR test. (**J**) Quantification of freezing time percentage in the contextual fear conditioning test. *n* = 7 per group. 3 m: 3 m young controls, 12 m: 12 m chow-fed controls, 12 m + HFD: 12-month-old HFD-fed mice. Data are presented as mean ± SEM. ns, not significant; * *p* < 0.05, ** *p* < 0.01, **** *p* < 0.0001. (Unpaired *t* test in (**B**). Ordinary one-way ANOVA in (**F**,**G**,**I**,**J**). Two-way ANOVA in (**D**,**E**)).

**Figure 2:**
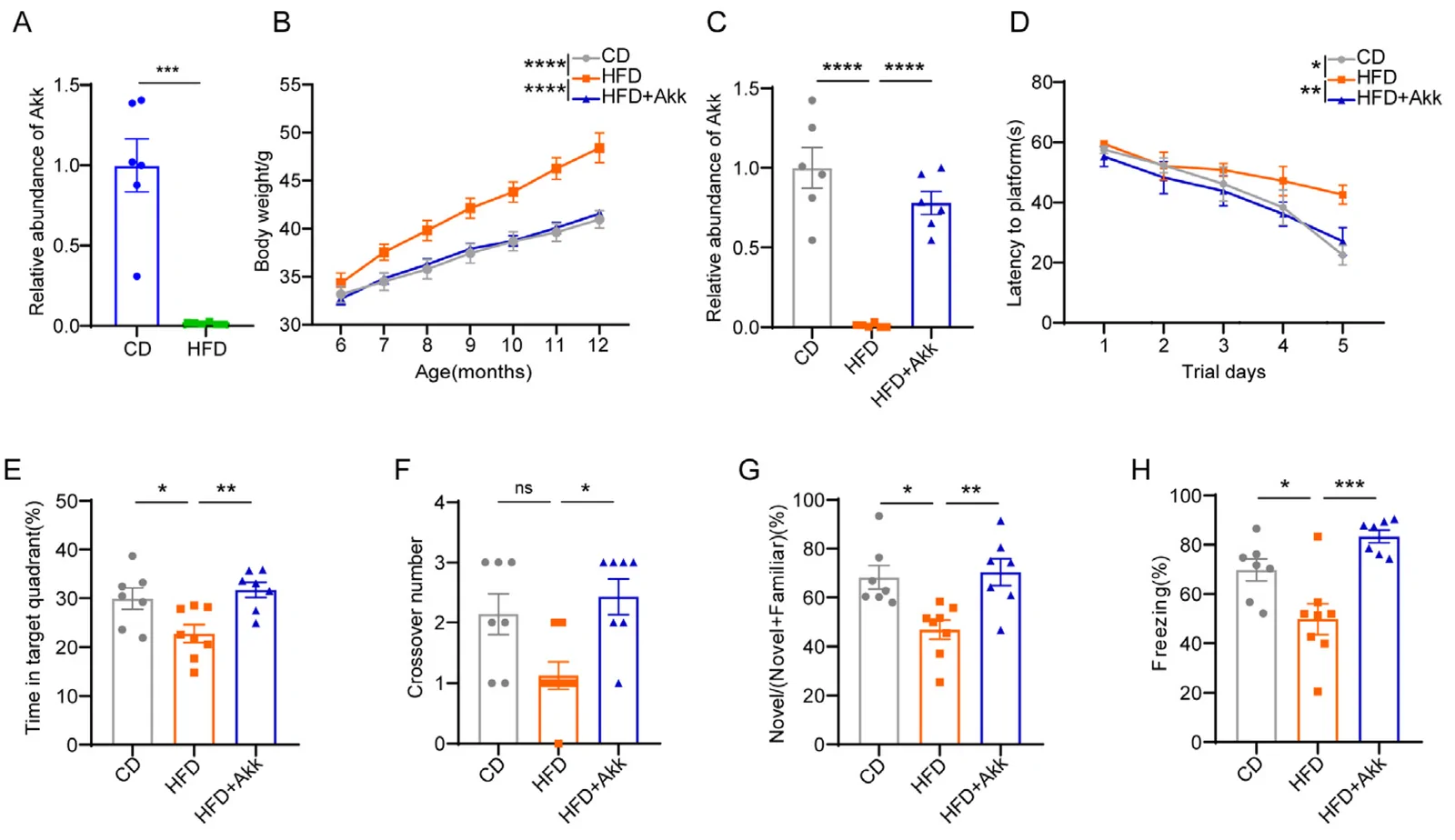
Effects of Akk supplementation on body weight and cognitive function in high-fat diet-fed middle-aged mice (**A**) Relative abundance of fecal Akk detected by qPCR in normal chow diet (CD) and high-fat diet (HFD) mice. *n* = 6 per group. (**B**) Body weight curves of CD, HFD, and HFD + Akk mice during the 6-month midlife intervention (6 to 12 months of age). *n* = 7–8 per group. (**C**) Relative abundance of fecal Akk across the three experimental groups. *n* = 7–8 per group. (**D**) Escape latency across the 5-day acquisition phase of the MWM test in the three groups. *n* = 7–8 per group. (**E**,**F**) Quantification of the percentage of time spent in the target quadrant (**E**) and the number of platform crossings (**F**) in the MWM spatial probe trial. *n* = 7–8 per group. (**G**) Quantification of the novel object exploration time percentage in the NOR test. *n* = 7–8 per group. (**H**) Quantification of freezing time percentage in the contextual fear conditioning test. *n* = 7–8 per group. CD, control diet group; HFD, high-fat diet group; HFD + Akk, high-fat diet with Akk supplementation group. Data are presented as mean ± SEM. * *p* < 0.05, ** *p* < 0.01, *** *p* < 0.001, **** *p* < 0.0001. (Unpaired *t* test in (**A**). Ordinary one-way ANOVA in (**C**,**E**,**G**,**H**). Kruskal–Wallis test in (**F**). Two -way ANOVA in (**B**,**D**)).

**Figure 3:**
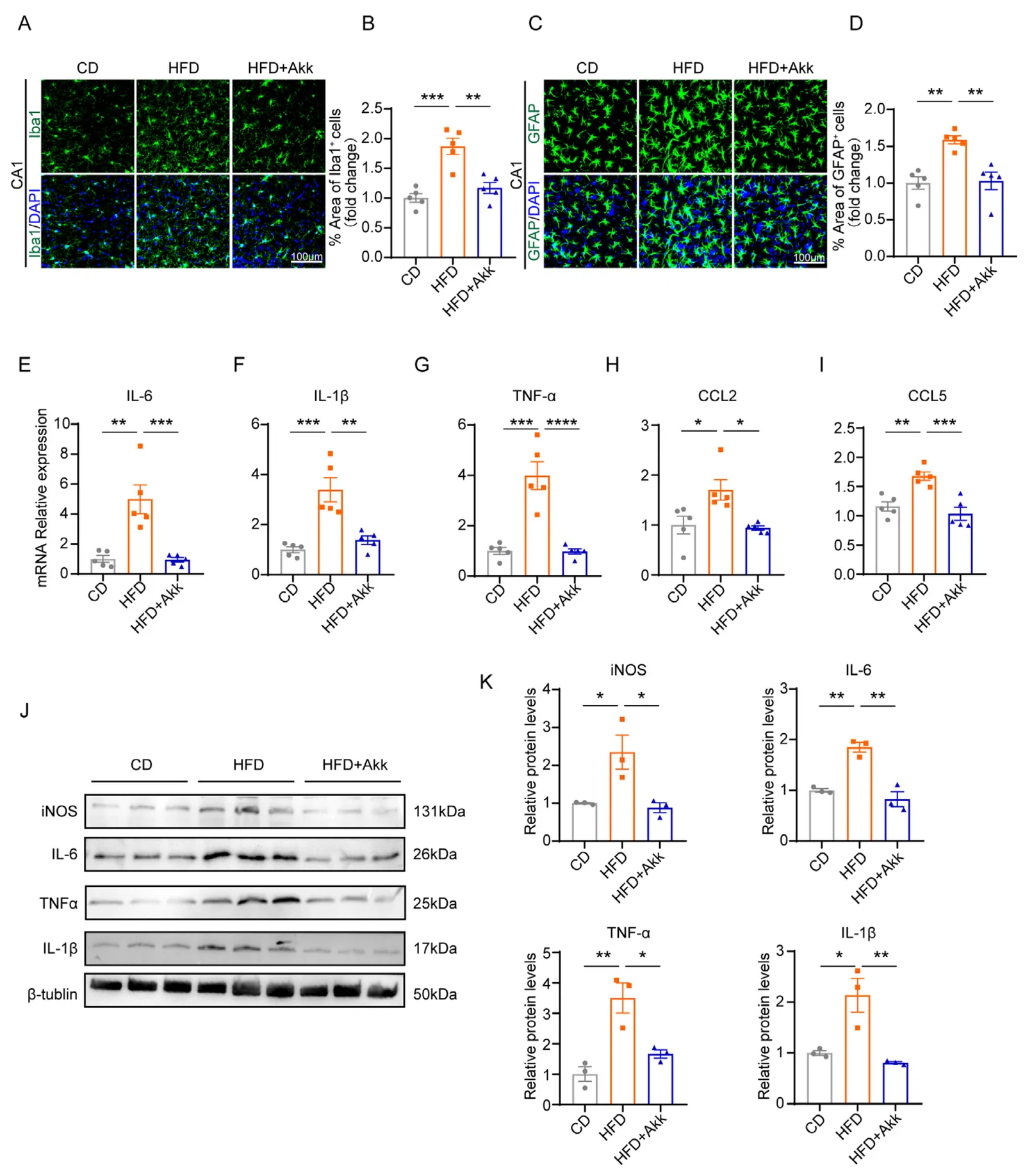
Akk reduces hippocampal neuroinflammation in HFD-fed mice. (**A**) Representative immunofluorescence images of Iba1 (green, microglia-specific marker) staining in the hippocampal CA1 region, with nuclei counterstained with DAPI (blue). Scale bar = 100 μm. (**B**) Quantitative analysis of the percentage of Iba1-positive area, presented as fold change relative to the CD group. n = 5 per group. (**C**) Representative immunofluorescence images of GFAP (green, astrocyte-specific marker) staining in the hippocampus CA1 region, with nuclei counterstained with DAPI (blue). Scale bar = 100 μm. (**D**) Quantitative analysis of the percentage of GFAP-positive area, presented as fold change relative to the CD group. *n* = 5 per group. (**E**–**I**) Relative mRNA expression levels of proinflammatory cytokines and chemokines in the hippocampus, including IL-6 (**E**), IL-1β (**F**), TNF-α (**G**), CCL2 (**H**) and CCL5 (**I**), detected by qPCR. (**J**,**K**) Representative Western blot bands and quantitative analysis of of inflammation-associated proteins, including iNOS, IL-6, TNF-α and IL-1β, with β-tubulin as the loading control. n = 3 per group. CD, control diet group; HFD, high-fat diet group; HFD + Akk, high-fat diet with Akk supplementation group. Data are presented as mean ± SEM. * *p* < 0.05, ** *p* < 0.01, *** *p* < 0.001, **** *p* < 0.0001. (Ordinary one-way ANOVA in (**B**,**D**,**E**–**I**,**K**)).

**Figure 4:**
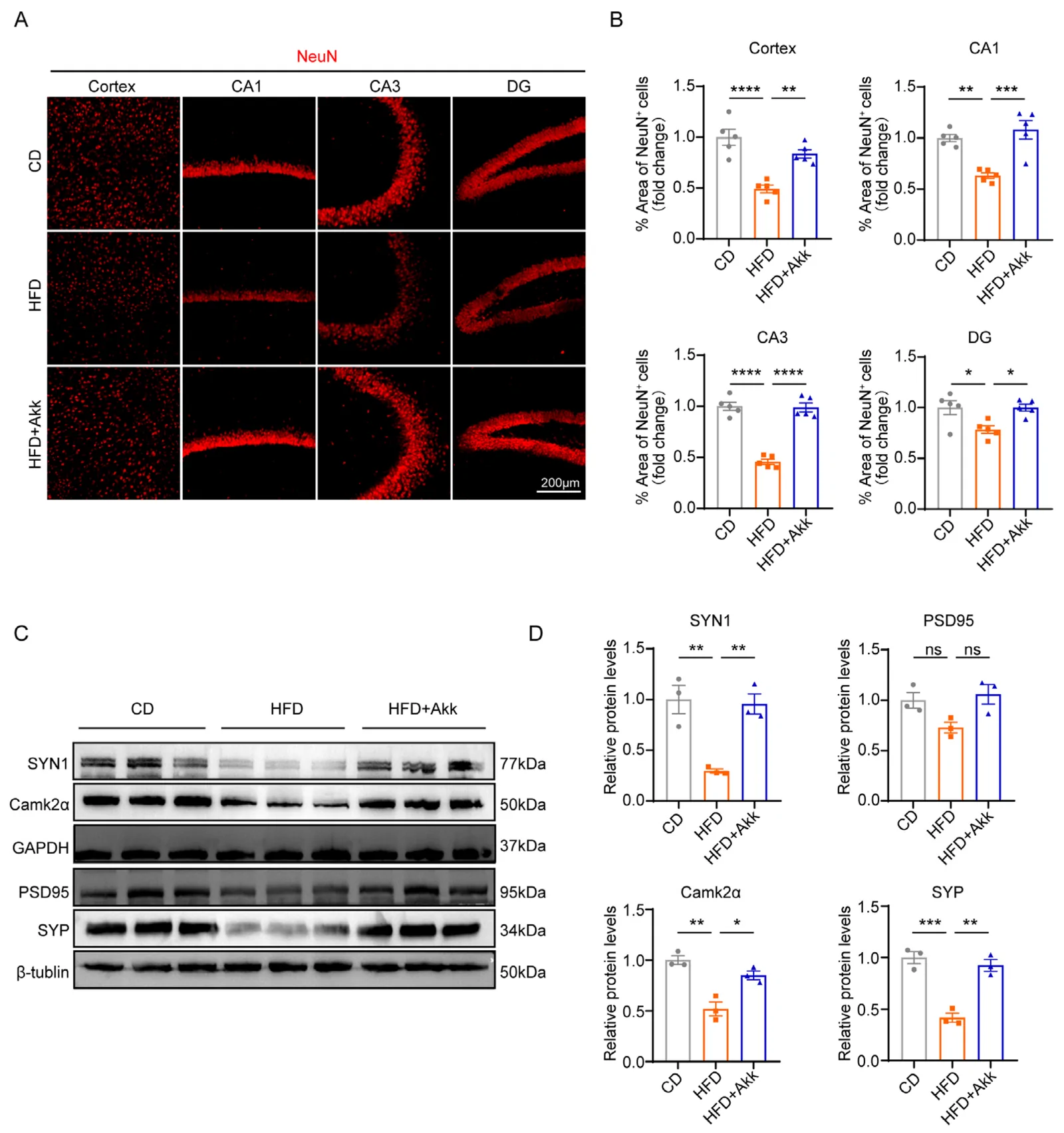
Akk preserves neuronal survival and synaptic integrity in HFD-fed mice. (**A**) Representative immunofluorescence images of NeuN (red, a mature neuron-specific marker) staining in the cerebral cortex and hippocampal subregions (CA1, CA3, DG). (**B**) Quantitative analysis of the percentage of NeuN-positive area in the cortex, CA1, CA3 and DG regions, presented as fold change relative to the CD group. *n* = 5 per group. (**C**) Representative Western blot bands of synaptic-associated proteins, including SYN1, PSD95, Camk2α and SYP, with β-tubulin/GAPDH as the loading control. (**D**) Quantitative analysis of relative expression levels of SYN1, PSD95, Camk2α and SYP in the hippocampus. *n* = 3 per group. CD, control diet group; HFD, high-fat diet group; HFD + Akk, high-fat diet with Akk supplementation group. Data are presented as mean ± SEM. (Ordinary one-way ANOVA in (**B**,**D**)). * *p* < 0.05, ** *p* < 0.01, *** *p* < 0.001, **** *p* < 0.0001.

**Figure 5:**
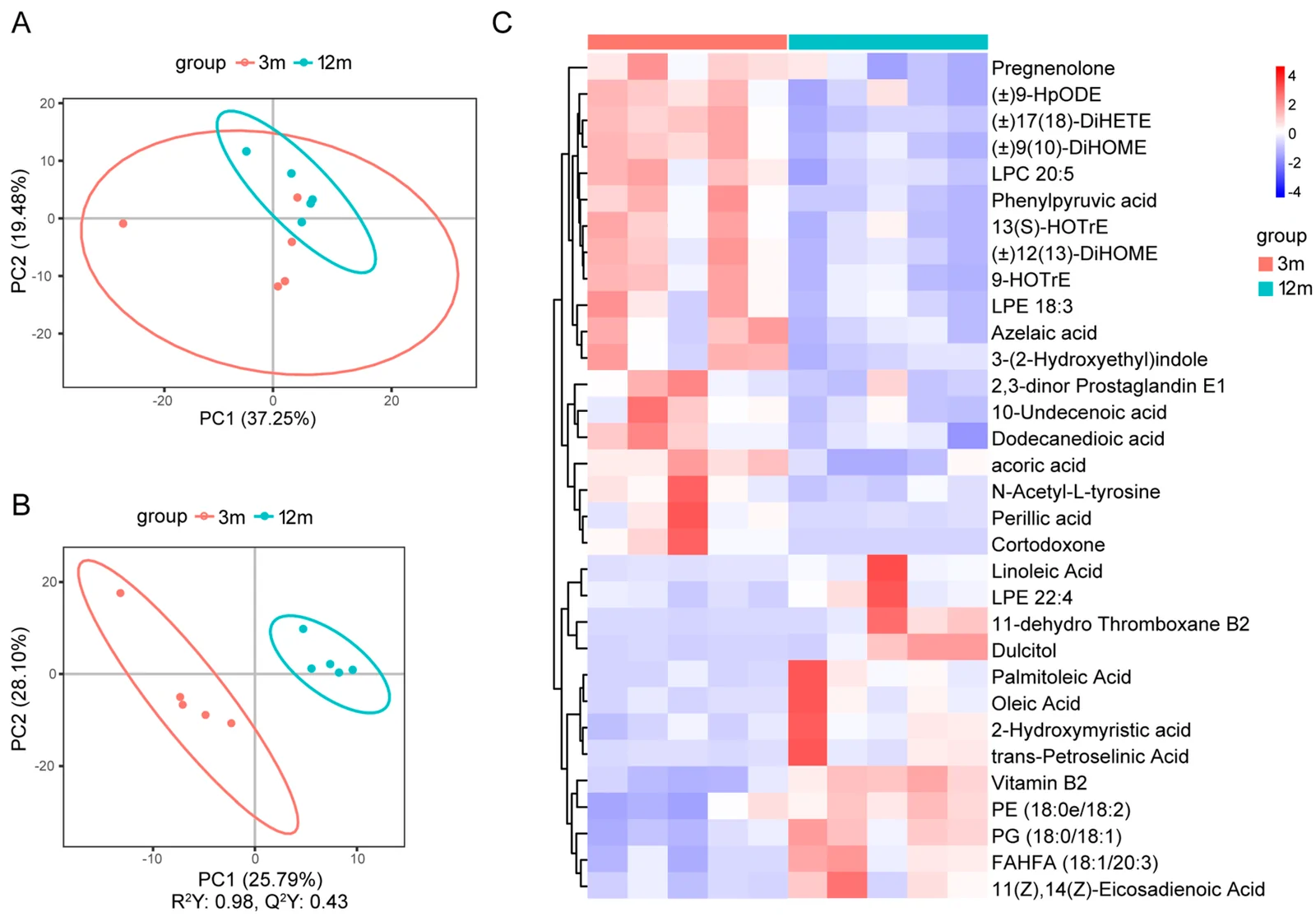
Multivariate statistical analysis and hierarchical clustering heatmap of serum metabolomes in young and middle-aged mice. (**A**) Unsupervised PCA score plot of serum metabolomes from the 3-month-old young group (3 m) and 12-month-old middle-aged group (12 m). PC1 and PC2 explain 37.25% and 19.48% of the total variance, respectively. Ellipses represent 95% confidence intervals of the samples. (**B**) Supervised PLS-DA score plot of serum metabolomes between the two groups, with model parameters R^2^Y = 0.98 and Q^2^Y = 0.43. Ellipses represent 95% confidence intervals of the samples. (**C**) Hierarchical clustering heatmap of significantly differential metabolites. Rows indicate individual metabolites, and columns indicate individual samples. *n* = 5 per group. The color gradient from blue to red corresponds to increasing relative abundance of metabolites. The top color bar denotes sample grouping (red: 3-month-old group; cyan: 12-month-old group).

**Table 1:** Detailed composition of the high-fat diet per 10 kg of product.

Component	Amount (g)
Vitamin Tested Casein	2580
L-Cystine	38.8
Maltodextrin	2000
Sucrose	889
Alphacel Non-Nutritive Bulk	646
Soybean Oil	323
Lard	3166
AIN-76 Mineral Mix	130
Dicalcium Phosphate	168
Potassium Citrate	213
Calcium Carbonate	71
Vitamin Mix	129
Choline Bitartrate	26
Dye	2.5

**Table 2:** Antibodies and experimental conditions used for Western blotting and immunofluorescence staining.

Reagent or Resource	Source	Identifier
NeuN Mouse Monoclonal Antibody	Cell Signaling Technology	Cat: #94403; RRID: AB_2904530
PSD95 Rabbit Monoclonal Antibody	Cell Signaling Technology	Cat: #3450; RRID: AB_2292883
Synapsin-1 Rabbit Monoclonal Antibody	Cell Signaling Technology	Cat: #5297; RRID: AB_2616578
CaMKII-alpha Mouse Monoclonal Antibody	Cell Signaling Technology	Cat: #50049; RRID: AB_2721906
Iba1 Rabbit Polyclonal Antibody	Wako	Cat: #01919741; RRID: AB_839504
GFAP Rabbit Polyclonal Antibody	Abcam	Cat: #ab7260; RRID: AB_305808
iNOS Rabbit Monoclonal Antibody	Abcam	Cat: #ab178945; RRID: AB_2861417
TNF-α Rabbit Monoclonal Antibody	Cell Signaling Technology	Cat: #11948; RRID: AB_2687962
IL-6 Rabbit Monoclonal Antibody	ABclonal	Cat: #A27935; RRID: AB_3751225
IL-1β Rabbit Monoclonal Antibody	ABclonal	Cat: #A22257; RRID: AB_3698196
β-Tubulin Rabbit Monoclonal Antibody	ABclonal	Cat: #A12289; RRID: AB_2861647
GAPDH Mouse Monoclonal Antibody	Proteintech	Cat: #60004-1-Ig; RRID: AB_2107436
Goat Anti-Rabbit IgG (H + L) Secondary Antibody, Alexa Fluor 488	Invitrogen	Cat: #A11094; RRID: AB_221544
Goat Anti-Mouse IgG (H + L) Secondary Antibody, Alexa Fluor 594	Invitrogen	Cat: #A11005; RRID: AB_2534073
Goat anti-Rabbit IgG (H + L) Secondary Antibody, HRP	Invitrogen	Cat: #31460; RRID: AB_ 228341
Goat anti-Mouse IgG (H + L) Secondary Antibody, HRP	Invitrogen	Cat: #31430; RRID: AB_ 228307

## Data Availability

All original data generated in this work are accessible within the main article. Additional requests for information may be addressed to the corresponding author.

## Abbreviations

HFD	High-fat diet
Akk	*Akkermansia muciniphila*
LBM	Lean body mass
CD	chow diet
MWM	Morris water maze
NOR	Novel object recognition
Iba1	Ionized calcium-binding adapter molecule 1
GFAP	Glial fibrillary acidic protein
IL-6	Interleukin-6
IL-1β	Interleukin-1β
TNF-α	Tumor necrosis factor-α
CCL2	C-C motif chemokine ligand 2
CCL5	C-C motif chemokine ligand 5
iNOS	Tnducible nitric oxide synthase
DAPI	4′,6-diamidino-2-phenylindole
Syn1	Synapsin I
PSD95	Postsynaptic density protein 95
CaMKIIα	Calcium/calmodulin-dependent protein kinase II α
SYP	Synaptophysin
PCA	Principal component analysis
PC1	The first principal component
PC2	The second principal component
PLS-DA	Partial least squares-discriminant analysis
VIP	Variable importance in projection
PG	Phosphatidylglycerol
PE	Phosphatidylethanolamine
